# When Can Predictive Brains be Truly Bayesian?

**DOI:** 10.3389/fpsyg.2012.00406

**Published:** 2012-11-07

**Authors:** Mark Blokpoel, Johan Kwisthout, Iris van Rooij

**Affiliations:** ^1^Donders Institute for Brain, Cognition and Behaviour, Radboud University NijmegenNijmegen, Netherlands

It is thus a major virtue of the hierarchical predictive coding account that it effectively implements a computationally tractable version of the so-called Bayesian Brain Hypothesis. (Clark, in press)

It seems by now common wisdom that a brain organized according to the principles of hierarchical predictive coding is a brain that is capable of efficiently performing full-blown Bayesian inferences. The idea is not only common, but also of great significance, as it suggests that the hierarchical predictive coding framework may provide a neurally plausible and computationally feasible bridge between theories of neural functioning (Friston, 2005) and theories of cognitive functioning (Chater and Manning, 2006; Baker et al., 2009).

But can predictive brains really be the same as Bayesian brains? Or is the claim merely an informal or imprecise shorthand for something which is formally and factually false? We address these questions by reconsidering the formal specifications of the theory of hierarchical predictive coding, as put forth by Friston (2002, 2005).

In the hierarchical predictive coding framework, it is assumed that the brain represents the statistical structure of the world at different levels of abstraction by maintaining different causal models that are organized on different levels of a hierarchy, where each level obtains input from its subordinate level. In a feed-backward chain, predictions are made for the level below. The error between the model’s predicted input and the observed (for the lowest level) or inferred (for higher levels) input at that level is used (a) in a feed-forward chain to estimate the causes at the level above and (b) to reconfigure the causal models for future predictions. Ultimately, the system stabilizes when it has minimized the overall prediction error.

Here we will focus on (a) the cause estimation step in the feed-forward chain. We will argue that the predictive coding framework does not yet satisfactorily specify how this step can be *both* Bayesian *and* computationally tractable. In the Bayesian interpretation of predictive coding (Friston, 2002) estimating the causes comes down to finding the most probable causes *v_m_* given the input *u* for that level and the current model parameters θ:

$$v_{m}=\arg\;\max_{v}\mathrm{Pr}\left(v|u;\text{θ}\right)$$

Given that *v_m_* has maximum *a posteriori* probability (MAP), the idea that predictive coding implements Bayesian inference seems to hinge on this step. The idea that hierarchical predictive coding implements *tractable* Bayesian inference in turn hinges on the presumed existence of a tractable computational method for estimating *v_m_*. Given that it is known that computing MAP—whether exactly or approximately—is computationally intractable for arbitrary causal structures (Shimony, 1994; Abdelbar and Hedetniemi, 1998; Kwisthout, 2011), the existence of a tractable method crucially depends on the structural properties of the brain’s causal models (Kwisthout et al., 2011).^1^

At present, the hierarchical predictive coding framework does not yet make stringent commitments as to the nature of the causal models that the brain can represent. Hence, contrary to suggestions by Clark (in press), the framework does not yet have the virtue that it effectively implements tractable Bayesian inference. At this point in time three mutually exclusive options remain open: either predictive coding does not implement Bayesian inference, or predictive coding is not tractable, or the theory of hierarchical predictive coding is enriched by specific assumptions about the structure of the brain’s causal models.

Assuming that one is committed to the Bayesian Brain Hypothesis, the first two options are out and the third is the only one remaining. Formal analyses expanding on this option are beyond the scope of this commentary (see e.g., Blokpoel et al., 2010; van Rooij et al., 2011), but Table 1 qualitatively sketches the space of causal models that could (or could not) yield tractable Bayesian cause estimation. We will discuss the viability of the options in more detail below.

**Table 1:** **For which types of causal models do there exist methods for cause estimation that are both tractable and Bayesian**?

Structure of causal models	Method used for cause estimation	Bayesian	Tractable
Simple	Heuristic	Yes	Yes
	Approximate	Yes	Yes

Intermediate	Heuristic	Maybe	Yes
	Approximate	Yes	Maybe

Unconstrained	Heuristic	No	Yes
	Approximate	Yes	No

To start, causal models could be assumed to be quite simple, e.g., having high degrees of statistical independencies of variables. In this case, it may be that heuristic methods, such as those based on gradient ascent (Friston, 2002, p. 13) or a Kalman filter (Rao and Ballard, 1999), yield tractable Bayesian cause estimation. Let’s assume that it does. Then, of course, also tractable approximation methods exist for those simple structures—the heuristics themselves being a case in point. Note, however, that a commitment to such simple causal models may limit the scope of the predictive coding theory to simple or low-level forms of perception and cognition. After all, higher-order causal reasoning—such as occurs, for instance, in Theory of Mind (Kilner et al., 2007)—seems to presuppose quite sophisticated causal structures containing complex statistical interdependencies (see Figure 1 for an illustration; cf. Uithol et al., 2011). Complex causal models can allow for rugged probability landscapes of different possible causes and heuristic methods can get stuck in local optima that may be arbitrarily far off from the true Bayesian (i.e., MAP) solution. For complex causal structures, heuristics are thus not guaranteed to do anything remotely like approximating Bayesian inference.

**Figure 1:**
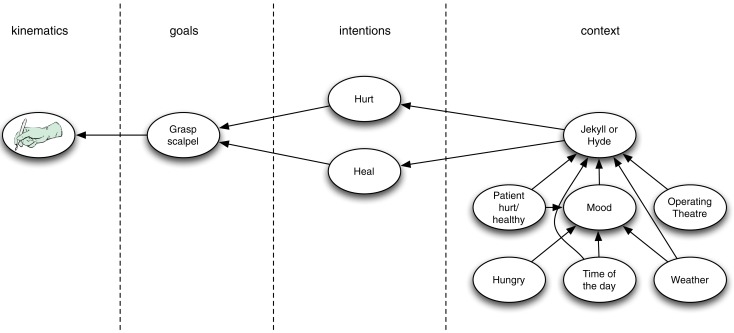
**An illustration of a hierarchy with higher level complex causal models**. The illustration builds on the Jekyll and Hyde example used by Kilner et al. (2007). Kilner et al. assumed four different levels and simple mappings between the levels. For example, if at the higher level one infers that the person grasping the scalpel is Dr. Jekyll (or Mr. Hyde) then at the lower level one predicts the intention is to heal (or to hurt). The Figure illustrates that at higher levels of the hierarchy the causal models within a level can become quite complex. Whether one infers that the person is Jekyll or Hyde can depend on a myriad of interconnected variables, such as the present location, the health status of the patient, the weather, and the person’s mood. Note that this complexity cannot be dissolved by decomposing the complex causal model into simple causal models at higher levels of the hierarchy, because complex models cannot generally be so decomposed. So it seems that if one wants to use the hierarchical predictive coding framework to explain high-level cognition, then complex models within levels are required.

Given that the hierarchical predictive coding framework seems to aspire spanning all levels of cognitive functioning, it probably does not want to commit to simple causal models. The other extreme—i.e., that the brain’s causal models are structurally unconstrained—is also excluded. As explained above, it follows from known intractability results for approximating MAP (Shimony, 1994; Abdelbar and Hedetniemi, 1998; Kwisthout, 2011) that such a brain cannot implement tractable Bayesian inference. We are thus left with the intermediate option: The causal models represented by the brain can be complex but not arbitrarily so. Given that the exact nature of this causal complexity will determine whether or not a hierarchical predictive coding architecture can implement tractable Bayesian inference, it seems vital for the viability of the marriage between the predictive coding framework and the Bayesian Brain Hypothesis to identify exactly what this nature is.

There is a strong appeal to the Bayesian Brain Hypothesis, as well as to the hypothesis that the brain implements cognition via hierarchical predictive coding. Given that the statistics of the world do not seem to be arbitrarily complex, it is conceivable that the brain has evolved specifically those constraints on its causal models that afford tractable Bayesian inference via hierarchical predictive coding. The open question remaining is what those constraints could possibly be. This question is particularly pressing, yet non-trivial to answer, if the hierarchical predictive coding account aims to apply to all levels of perception and cognition.

## Footnotes

^1^We note that, for arbitrary causal structures, having the prediction and the prediction error in the input when estimating *v_m_* does not make this estimation computationally tractable.
